# Isolated Cardiac Sarcoidosis With Marfan Syndrome Following Cardiovascular Surgery: A Case Report

**DOI:** 10.7759/cureus.110584

**Published:** 2026-06-10

**Authors:** Tatsuro Tasaka, Toru Miyoshi, Masaki Kinoshita, Makoto Saito

**Affiliations:** 1 Department of Cardiology, Kitaishikai Hospital, Ozu, JPN; 2 Department of Cardiology, Pulmonology, Hypertension and Nephrology, Ehime University School of Medicine, Toon, JPN

**Keywords:** connective tissue disorder, heart failure with reduced ejection fraction, inflammatory cardiomyopathy, isolated cardiac sarcoidosis, marfan syndrome, multimodality imaging, postoperative heart failure

## Abstract

A 55-year-old male patient with Marfan syndrome presented with exertional dyspnea. The patient had undergone aortic valve and ascending aortic replacement and experienced two admissions for congestive heart failure. His thoracic echocardiography revealed severe left ventricular systolic dysfunction with regional wall motion abnormalities and an apical aneurysm. Meanwhile, coronary angiography showed no significant stenosis. Cardiac magnetic resonance imaging demonstrated wall thinning and late gadolinium enhancement (LGE) in the apical, inferior, and inferolateral segments, corresponding to focal-on-diffuse myocardial uptake on ^18^F-fluorodeoxyglucose positron emission tomography/computed tomography. No extracardiac sarcoid lesions were identified. Moreover, right ventricular endomyocardial biopsy was nondiagnostic. Based on the multimodality imaging and laboratory findings, a clinical diagnosis of isolated cardiac sarcoidosis was established according to current guidelines. The patient underwent cardiac resynchronization therapy with defibrillator implantation followed by corticosteroid therapy, with no subsequent heart failure events. This case highlights the importance of considering inflammatory cardiomyopathy for postoperative heart failure in patients with Marfan syndrome.

## Introduction

Cardiac sarcoidosis (CS) is an inflammatory cardiomyopathy with heterogeneous clinical manifestations and variable disease severity [1]. The diagnosis is often challenging, particularly in the absence of histological confirmation, and relies heavily on multimodality imaging such as cardiac magnetic resonance imaging and 18F-fluorodeoxyglucose positron emission tomography/computed tomography (FDG-PET/CT) [2,3]. Echocardiography may reveal segmental wall thinning with high specificity, although its sensitivity is limited [4]. Cardiac magnetic resonance imaging provides a detailed structural assessment, while FDG-PET/CT is highly sensitive for detecting active inflammation, often demonstrating increased uptake in early-stage disease. In the present literature, FDG-PET/CT has been reported to show a sensitivity of approximately 81-84% and a specificity of approximately 82% [5,6]. However, its specificity is limited due to physiological myocardial uptake, and careful clinical evaluation with exclusion of alternative diagnoses remains essential.

Previous studies have suggested that a subset of patients with Marfan syndrome may develop myocardial dysfunction, including primary myocardial involvement and progressive ventricular dysfunction following cardiovascular surgery [7,8]. A recent review has further highlighted cardiomyopathy as an increasingly recognized manifestation of Marfan syndrome and emphasized the potential contribution of both primary myocardial disease and postoperative ventricular dysfunction [9]. However, the coexistence of Marfan syndrome and CS is extremely rare, and only a limited number of cases have been reported [10].

We report a case of isolated cardiac sarcoidosis (iCS) diagnosed by multimodality imaging in a patient with Marfan syndrome.

## Case presentation

A 55-year-old male patient with a history of Marfan syndrome presented with exertional dyspnea. The patient had undergone aortic valve replacement and ascending aortic replacement in his 20s and had been hospitalized twice for acute decompensated heart failure approximately four years before the current admission. Physical findings supporting the diagnosis of Marfan syndrome included a reduced upper-to-lower segment ratio and an increased arm span-to-height ratio. Detailed information regarding revised Ghent criteria and FBN1 genetic testing was not available retrospectively.

According to records from the previous hospital, left ventricular systolic function had been preserved without regional wall motion abnormalities until approximately nine years before the current admission. Eight years before admission, transthoracic echocardiography revealed a reduced left ventricular ejection fraction (LVEF) of 45% with left ventricular dilation (left ventricular end-diastolic diameter (LVEDD): 59 mm; left ventricular end-systolic diameter (LVESD): 45 mm) and inferior wall motion abnormalities.

During hospitalization four years before the current admission, transthoracic echocardiography demonstrated further left ventricular dilation (LVEDD: 68 mm; LVESD: 58 mm) and worsening systolic dysfunction with an LVEF of 30%, accompanied by diffuse hypokinesis that was most prominent in the inferior and inferolateral walls. Given his surgical history, these findings were attributed to postoperative cardiomyopathy.

After these hospitalizations, the patient was referred to our hospital and followed regularly under guideline-directed medical therapy. Despite optimal medical treatment, his symptoms gradually worsened over time, progressing from New York Heart Association (NYHA) functional class II to class III prior to the current admission.

On admission, the patient’s blood pressure and heart rate were 78/39 mmHg and 61 beats/min, respectively. The hypotension was considered to reflect a chronic low-output state associated with severe left ventricular systolic dysfunction rather than overt cardiogenic shock. Electrocardiography revealed right axis deviation, atrial fibrillation with nonspecific intraventricular conduction delay (Figure 1A).

**Figure 1:**
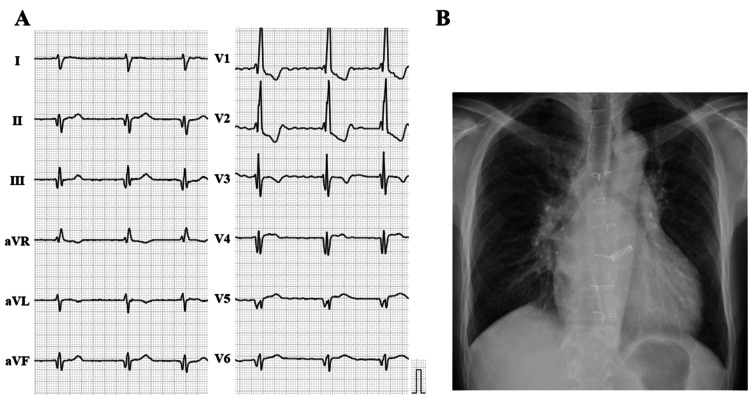
Electrocardiogram and chest radiograph (A) Twelve-lead electrocardiogram showing right axis deviation, atrial fibrillation, and nonspecific intraventricular conduction delay; (B) Chest radiograph revealing cardiomegaly with preserved costophrenic angles.

In addition, chest radiography showed cardiomegaly with preserved costophrenic angles (Figure 1B). Transthoracic echocardiography demonstrated inferolateral wall akinesis, diffuse hypokinesis, LVEF of 27%, left ventricular dilation (LVEDD: 74 mm; LVESD: 65 mm), and apical aneurysm (Figure 2A; Videos 1-3).

**Figure 2:**
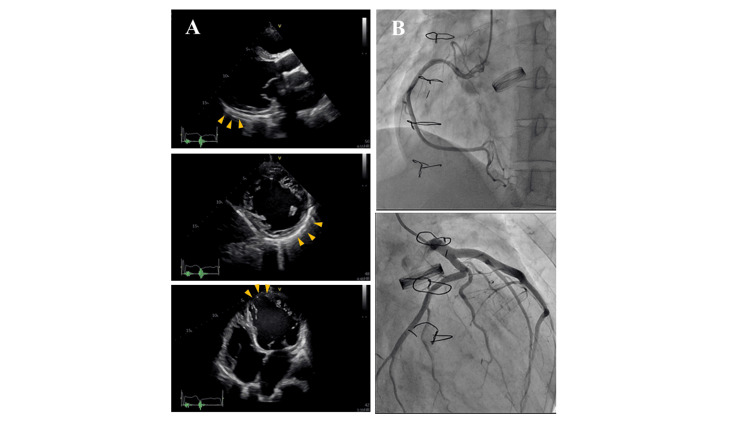
Coronary angiography and transthoracic echocardiography (A) Transthoracic echocardiography showing inferior and inferolateral wall akinesis and an apical aneurysm (arrows); (B) Coronary angiography showing no significant stenosis in the major coronary arteries.

**Video 1:** Transthoracic echocardiography (parasternal long-axis view) showing left ventricular dilation and reduced systolic function

**Video 2:** Transthoracic echocardiography (parasternal short-axis view) demonstrating inferolateral wall akinesis with diffuse hypokinesis

**Video 3:** Transthoracic echocardiography (apical four-chamber view) showing apical aneurysm formation and reduced left ventricular systolic function

B-type natriuretic peptide was elevated at 1,013 pg/mL (normal: <18.6 pg/mL) and troponin I was 17.9 pg/mL (normal: <26.2 pg/mL).The patient was treated with intravenous dobutamine at 2 μg/kg/min and intravenous furosemide at 20 mg/day for acute decompensated heart failure.

Coronary angiography performed after stabilization revealed no significant coronary stenosis (Figure 2B). However, cardiac magnetic resonance imaging showed thinning and wall motion reduction of the apical, inferior, and inferolateral segments, with late gadolinium enhancement (LGE) in the corresponding regions (Figure 3) consistent with a nonischemic pattern.

**Figure 3:**
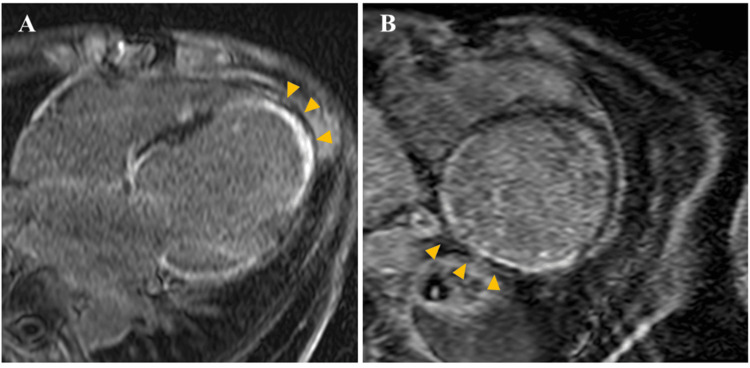
Cardiac magnetic resonance imaging (A) Long-axis view showing late gadolinium enhancement in the apical segment with a nonischemic distribution (arrows); (B) Short-axis view showing late gadolinium enhancement in the inferior and inferolateral walls (arrows).

Considering that the findings, including reduced LVEF, regional wall motion abnormalities, ventricular wall thinning, and LGE on cardiac magnetic resonance imaging, were suggestive of CS, additional testing was performed according to guideline recommendations [1]. Laboratory testing was notable only for elevated lysozyme levels, while other sarcoidosis-related markers remained within normal limits (Table 1).

**Table 1:** Additional laboratory investigations performed for the evaluation of suspected cardiac sarcoidosis sIL-2R: soluble interleukin-2 receptor; ACE: angiotensin-converting enzyme.

Parameter	Patient value	Reference range
Lysozyme	17.0 µg/mL	5.0–10.0 µg/mL
sIL-2R	470 U/mL	122–496 U/mL
ACE	11.8 U/L	7–25 U/L
Calcium	9.2 mg/dL	8.8–10.2 mg/dL

FDG-PET/CT was performed following a preparation protocol that combined a low-carbohydrate diet with 18-hour fasting. The imaging revealed focal-on-diffuse myocardial uptake corresponding to LGE distribution (Figure 4A), with a maximum standardized uptake value (SUVmax) of 13.

**Figure 4:**
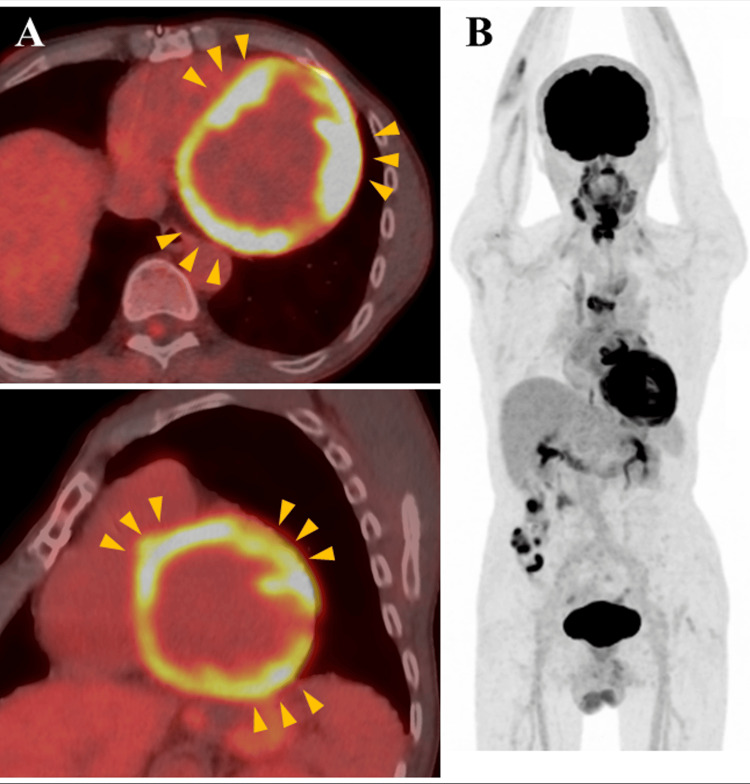
¹⁸F-fluorodeoxyglucose positron emission tomography/computed tomography (FDG-PET/CT) (A) FDG-PET/CT showing abnormal myocardial uptake corresponding to the LGE distribution, with focal myocardial uptake on diffuse background activity (arrows); (B) Whole-body FDG-PET showing no clinically significant extracardiac uptake suggestive of sarcoidosis; mild uptake in the ascending colon was considered physiologic.

No clinically significant extracardiac uptake was identified. Mild uptake was observed in the ascending colon; subsequent colonoscopy confirmed this finding to be physiologic (Figure 4B). Unfortunately, right ventricular endomyocardial biopsy revealed no non-caseating granulomas.

Based on the absence of extracardiac sarcoidosis findings and fulfillment of four of the five major cardiac criteria - specifically, reduced LVEF, regional wall motion abnormalities with ventricular wall thinning, LGE on cardiac magnetic resonance imaging, and abnormal myocardial FDG uptake on PET/CT - the patient was considered to fulfill the diagnostic criteria for clinically diagnosed iCS according to the Japanese Circulation Society guidelines [1]. Although pathological confirmation by endomyocardial biopsy was not obtained, the multimodality imaging findings supported the diagnosis of iCS, and no alternative diagnosis was considered more likely based on the available clinical and imaging findings.

Considering the patient's severely reduced LVEF, progressive heart failure, and the increased risk of ventricular arrhythmias associated with CS, cardiac resynchronization therapy with defibrillator (CRT-D) implantation was performed for primary prevention in accordance with current guideline recommendations.

Prednisolone therapy was subsequently initiated at 30 mg/day for four weeks and tapered by 5 mg every four weeks.

After six months, his follow-up FDG-PET/CT showed no significant interval change in myocardial uptake. However, the patient remained clinically stable without heart failure hospitalization during outpatient follow-up.

## Discussion

Previous studies have reported an association between Marfan syndrome and cardiomyopathy [7,8]. The term “Marfan cardiomyopathy” was first introduced in 1993 during the Symposium on Cardiovascular Aspects of Marfan Syndrome in Berlin, where Hetzer et al. noted that cardiomyopathy appeared to occur more frequently in patients with Marfan syndrome than in the general population [11]. In addition to aortic complications, abnormalities of fibrillin-1 have been associated with intrinsic myocardial dysfunction, reduced myocardial compliance, and ventricular remodeling. Such myocardial involvement may contribute to valvular insufficiency and the development of heart failure independent of aortic pathology. Moreover, postoperative cardiomyopathy has been reported in Marfan syndrome. In a series of patients who eventually underwent cardiac transplantation, all patients had a history of previous cardiovascular surgery. In addition, cases of sarcoidosis occurring in patients with Marfan syndrome have also been described [10]. Furthermore, CS is reportedly associated with other inherited cardiomyopathies. However, there is no evidence supporting a causal relationship or predisposition conferred by genetic cardiomyopathies [12]. According to a previous study, such coexistence is likely coincidental, representing overlapping phenotypes or diagnostic challenges rather than disease-specific interactions [12].

Therefore, in the present case, the coexistence of Marfan syndrome and iCS is best interpreted as an incidental combination of a genetic connective tissue disorder and an inflammatory cardiomyopathy, which complicated the diagnostic process. Because of the patient’s surgical history, postoperative cardiomyopathy was initially suspected.

However, the presence of distinct regional wall motion abnormalities in the absence of coronary stenosis indicated an alternative etiology. Previous reports of Marfan-associated or postoperative cardiomyopathy have mainly described left ventricular dilatation and systolic dysfunction rather than localized wall motion abnormalities or ventricular aneurysm formation. In the present case, progressive regional wall motion abnormalities, nonischemic LGE, and focal-on-diffuse FDG uptake appeared atypical for previously reported Marfan-associated cardiomyopathy. Therefore, alternative diagnoses, including inflammatory cardiomyopathy, should also have been considered rather than attributing the findings solely to postoperative cardiomyopathy.

Cardiac magnetic resonance imaging and FDG-PET/CT findings were supportive of CS according to current diagnostic criteria [2]. The diagnostic yield of endomyocardial biopsy for CS is approximately 20% and may be as low as 10% in patients with suspected iCS [13]. Therefore, biopsy-negative findings do not exclude the diagnosis, particularly when multimodality imaging findings fulfill established diagnostic criteria. Advances in multimodality imaging have significantly improved diagnostic capabilities, particularly when histological confirmation is difficult [3].

Although T2-weighted imaging, native T1/T2 mapping, and extracellular volume analysis may provide additional information regarding myocardial inflammation and tissue characterization, these parameters were not acquired in the present case and therefore represent a limitation of the diagnostic evaluation.

Accordingly, current guidelines allow clinical diagnosis of CS based on imaging and clinical findings in biopsy-negative patients [14]. Prognosis does not significantly differ between histologically confirmed and clinically diagnosed CS. Thus, early clinical diagnosis with initiation of immunosuppressive therapy is recommended [14]. In patients with reduced left ventricular function or previous ventricular arrhythmias, device therapy, including implantable cardioverter-defibrillator or CRT-D, is essential, regardless of whether the diagnosis is histological or clinical [14,15].

Although follow-up FDG-PET/CT showed no significant interval improvement, the imaging findings and clinical course remained compatible with inflammatory cardiomyopathy. However, the lack of clear PET improvement limited the value of treatment response as supportive evidence for the diagnosis.

Although cardiomyopathy has been reported after cardiovascular surgery in patients with Marfan syndrome, the coexistence of Marfan syndrome and CS in this case was likely incidental. However, this case highlights the importance of careful diagnostic reassessment when progressive postoperative heart failure cannot be fully explained by previously reported postoperative or Marfan-associated cardiomyopathy. Because therapeutic strategies, particularly immunosuppression and device implantation, substantially differ from those for other cardiomyopathies, accurate diagnosis is clinically important.

This case has several limitations. First, histological confirmation was not obtained because right ventricular endomyocardial biopsy did not demonstrate non-caseating granulomas. As the biopsy was not guided by FDG-PET or electroanatomic mapping, sampling error cannot be excluded. Second, advanced tissue characterization by cardiac magnetic resonance imaging, including T2-weighted imaging, native T1/T2 mapping, and extracellular volume quantification, was not available. Third, follow-up FDG-PET/CT after corticosteroid therapy did not demonstrate a clear reduction in myocardial uptake. Although this finding does not exclude an inflammatory cardiomyopathy, it limits the extent to which treatment response can be considered supportive of the diagnosis. Finally, although the patient fulfilled the Japanese Circulation Society criteria for clinically diagnosed iCS, the absence of histological confirmation leaves a degree of diagnostic uncertainty.

## Conclusions

This case highlights the diagnostic challenge of differentiating postoperative cardiomyopathy from other causes of progressive left ventricular dysfunction in a patient with Marfan syndrome. Multimodality imaging, including cardiac magnetic resonance imaging and FDG-PET/CT, supported a clinical diagnosis of iCS according to contemporary diagnostic criteria, although residual diagnostic uncertainty remained because histological confirmation was not obtained and the treatment response was atypical. This case underscores the importance of careful diagnostic reassessment and considering a broader differential diagnosis in patients with progressive postoperative heart failure. In particular, progressive left ventricular dysfunction in patients with Marfan syndrome after cardiovascular surgery should prompt consideration of alternative diagnoses beyond postoperative or Marfan cardiomyopathy.

## References

[1] Terasaki F, Azuma A, Anzai T (2019). JCS 2016 guideline on diagnosis and treatment of cardiac sarcoidosis - digest version. Circ J.

[2] Ishimaru S, Tsujino I, Takei T (2005). Focal uptake on 18F-fluoro-2-deoxyglucose positron emission tomography images indicates cardiac involvement of sarcoidosis. Eur Heart J.

[3] Stjepanovic M, Markovic F, Milivojevic I (2024). Contemporary diagnostics of cardiac sarcoidosis: the importance of multimodality imaging. Diagnostics (Basel).

[4] Kato Y, Morimoto S (2008). Diagnosis and treatment of cardiac sarcoidosis (Article in Japanese). Jpn J Sarcoidosis Other Granulomatous Dis.

[5] Tang R, Wang JT, Wang L, Le K, Huang Y, Hickey AJ, Emmett L (2016). Impact of patient preparation on the diagnostic performance of 18F-FDG PET in cardiac sarcoidosis: a systematic review and meta-analysis. Clin Nucl Med.

[6] Aitken M, Chan MV, Urzua Fresno C (2022). Diagnostic accuracy of cardiac MRI versus FDG PET for cardiac sarcoidosis: a systematic review and meta-analysis. Radiology.

[7] Hetzer R, Siegel G, Delmo Walter EM (2016). Cardiomyopathy in Marfan syndrome. Eur J Cardiothorac Surg.

[8] Alpendurada F, Wong J, Kiotsekoglou A (2010). Evidence for Marfan cardiomyopathy. Eur J Heart Fail.

[9] Singh J, Wanjari A (2022). Cardiac complications in Marfan syndrome: a review. Cureus.

[10] Cobben NA, Lamers RJ, Drent M (1997). A manifestation of sarcoidosis in a woman with Marfan's syndrome. Sarcoidosis Vasc Diffuse Lung Dis.

[11] Hetzer R, Gehle P, Ennker J (1995). Morning panel session. Cardiovascular Aspects of Marfan Syndrome.

[12] Ebbinghaus H, Ueberham L, Husser-Bollmann D, Bollmann A, Laufs U, Dinov B (2023). Case report: four cases of cardiac sarcoidosis in patients with inherited cardiomyopathy-a phenotypic overlap, co-existence of two rare cardiomyopathies or a second-hit disease. Front Cardiovasc Med.

[13] Okada T, Kawaguchi N, Miyagawa M (2023). Clinical features and prognosis of isolated cardiac sarcoidosis diagnosed using new guidelines with dedicated FDG PET/CT. J Nucl Cardiol.

[14] Kitai T, Kohsaka S, Kato T (2025). JCS/JHFS 2025 guideline on diagnosis and treatment of heart failure. Circ J.

[15] Kusano K, Ishibashi K, Noda T (2021). Prognosis and outcomes of clinically diagnosed cardiac sarcoidosis without positive endomyocardial biopsy findings. JACC Asia.

